# Effects of coronatine elicitation on growth and metabolic profiles of *Lemna paucicostata* culture

**DOI:** 10.1371/journal.pone.0187622

**Published:** 2017-11-03

**Authors:** Jin-Young Kim, Hye-Youn Kim, Jun-Yeong Jeon, Dong-Min Kim, Yaoyao Zhou, Jae Soung Lee, Heayyean Lee, Hyung-Kyoon Choi

**Affiliations:** College of Pharmacy, Chung-Ang University, Seoul, Republic of Korea; National Research Council of Italy, ITALY

## Abstract

In this study, the effects of coronatine treatment on the growth, comprehensive metabolic profiles, and productivity of bioactive compounds, including phenolics and phytosterols, in whole plant cultures of *Lemna paucicostata* were investigated using gas chromatography-mass spectrometry (GC-MS) coupled with multivariate statistical analysis. To determine the optimal timing of coronatine elicitation, coronatine was added on days 0, 23, and 28 after inoculation. The total growth of *L*. *paucicostata* was not significantly different between the coronatine treated groups and the control. The coronatine treatment in *L*. *paucicostata* induced increases in the content of hydroxycinnamic acids, such as caffeic acid, isoferulic acid, *ρ*-coumaric acid, sinapic acid, and phytosterols, such as campesterol and β-sitosterol. The productivity of these useful metabolites was highest when coronatine was added on day 0 and harvested on day 32. These results suggest that coronatine treatment on day 0 activates the phenolic and phytosterol biosynthetic pathways in *L*. *paucicostata* to a greater extent than in the control. To the best of our knowledge, this is the first report to investigate the effects of coronatine on the alteration of metabolism in *L*. *paucicostata* based on GC-MS profiling. The results of this research provide a foundation for designing strategies for enhanced production of useful metabolites for pharmaceutical and nutraceutical industries by cultivation of *L*. *paucicostata*.

## Introduction

*Lemna paucicostata* (commonly known as duckweed) is a free-floating aquatic plant, which belongs to the Araceae family, and it is commonly found in ponds or in rice fields [1,2]. Because of high protein content, *Lemna* species are used as a source of livestock and fish feed [3]. Previous studies have reported that duckweed can play a positive role in ecologically and economically by removing toxic substances such as antimicrobials, boron, and benzotriazoles, from contaminated water resources [4–6]. It was also reported that metabolic profiling with *Lemna* species had been developed to routine use for early characterization of known mode of action and for the discovery of novel mode of action of various herbicides [7]. In addition, *L*. *paucicostata* extract has important pharmacological roles because of its antitumor and immunomodulatory activities [2].

Secondary metabolites present in plants are sources of pharmaceuticals, food components, flavors, and other industrial materials [8]. These metabolites have various functions; for example, they possess antitumor, antioxidant, and antiinflammatory activities [9]. However, neither the total extraction of these secondary metabolites from natural resources nor their total chemical synthesis is economically viable because of low productivities, high production cost, and various environmental factors [10,11]. The strategies that have been used to increase the production of medicinally valuable secondary metabolites include optimization of *in vitro* culture conditions, selection of high-producing cell lines, and precursor feeding and biotransformation [12]. In addition, elicitation in plant cell culture has been recognized as a useful strategy for enhancement of secondary metabolites [11,13]. Elicitation is one of the effective methods to improve secondary metabolites production in plant cell, tissue, and organ culture. Elicitation can be defined as induction of increased productions of secondary metabolites such as antibiotics by biotic (glucan polymers, glycoproteins, fungal cell wall materials) and abiotic (ultraviolet irradiation, heavy metals, and various chemicals) elicitors in plant cell, tissue, and organ cultures [14].

Coronatine, produced by pathovars of plant bacteria *Pseudomonas syringae*, is a molecular mimic of the isoleucine-combined form of jasmonic acid, which is a plant growth regulator and an elicitor that induces secondary metabolites in plants [15]. A previous study reported that coronatine was more effective in increasing flavonoid production than jasmonic acid and increased the stress resistance in cotton plants [16]. In addition, coronatine improved the antioxidant activity in soybean plants and, thereby, preserved their high photosynthetic performance [17]. Cucumber plants treated with coronatine were reported to have improved antioxidant defense system [18]. Furthermore, coronatine treatment enhanced the accumulation of taxane in *Taxus media* and *Corylus avellana* cell cultures [19,20].

Metabolomics has been used to study various plant cell cultures. Because the metabolome is a direct reflection of the existing functional state of a plant system, the levels of metabolites may change significantly during biochemical reactions [21]. Thus, metabolic profiling can be used as a powerful tool to investigate the metabolic response of plants to environmental perturbations, such as elicitor treatment, nutrient deficiency, high salinity, and temperature stress [22]. In previous studies, the metabolic profiles of seedling shoots and roots of two rice (*Oryza sativa* L.) cultivars in response to drought and high salinity stress were investigated by nuclear magnetic resonance (NMR) analysis [23], and the methyl jasmonate-induced defense response in *Centella asiatica* cells was revealed by changes in the metabolite profiles based on gas chromatography-mass spectrometry (GC-MS) and liquid chromatography-mass spectrometry (LC-MS) platforms [24]. In addition, a comparative GC-MS profiling was performed to examine the effects of methyl jasmonate and silver nitrate treatments on the growth and metabolic profiles of *L*. *paucicostata* [25].

Few studies have been conducted to investigate the effect of elicitation on the metabolic profiles of *L*. *paucicostata* and *Wolffia arrhiza* [25,26]. To the best of our knowledge, the effects of coronatine elicitation on the growth and metabolic profiles in whole plant cultures of *L*. *paucicostata* have not been examined. In this study, we investigated the effect of coronatine on the growth and metabolic profiles of the whole plants of *L*. *paucicostata* using GC-MS coupled with multivariate data analysis.

## Materials and methods

### Cultivation of whole plants of *L*. *paucicostata*

The *L*. *paucicostata* (PC-10605) plants (synonym *Lemna aequinoctialis* [27]) were obtained from the Korean Collection for Type Cultures (KCTC) (Biological Resource Center, Jeongeup, Republic of Korea) and grown under conditions of 25 ± 1°C temperature, 81 μmol/m^2^ s light intensity, and 16-h light:8-h dark day length in an incubator (NEX-202M, EYELA, Nexus Technologies, Seoul, Republic of Korea). The whole *L*. *paucicostata* plants were cultured on solid 1/2MS1BA medium (20 mL) containing half-strength Murashige and Skoog’s basal medium [28] supplemented with 1 mg/L benzylaminopurine (BA), 30 g/L sucrose, and 6 g/L Gelrite (Duchefa Biochemie, Haarlem, The Netherlands) on once every two weeks. The pH was adjusted to 5.8 prior to autoclaving at 121°C for 20 min. The liquid 1/2MS1BA medium was prepared as described above, but did not contain Gelrite. Thirty of *L*. *paucicostata* fronds (length: 3.5 mm, width: 2.5 mm, thinkness < 0.09 mm per one frond) were transferred to Erlenmeyer flasks filled with 100 mL of liquid 1/2MS1BA medium.

### Coronatine treatment

Thirty *L*. *paucicostata* plants were inoculated into Erlenmeyer flasks containing 100 mL of liquid 1/2MS1BA medium and were treated with 1 μM coronatine on day 0, day 23 (mid exponential stage), and day 28 (early stationary stage). The coronatine treatment time points of day 23 and 28 were determined from preliminary experiments based on total plant growth of *L*. *paucicostata* including meristem region and differentiated tissues (S1 Fig). The experiments were performed in four replicates. The plants, sampled at day 27 and 32, were freeze-dried using a freeze-drier (Bondiro, Ilshin Lab. Co., Ltd., Seoul, Republic of Korea) for 48 h and used for further analysis.

### Growth measurement

The number of the fronds was counted on days 0, 7, 14, 21, 27, 28, and 32, and the dry weight of the *L*. *paucicostata* cultivated in the presence of 1 μM coronatine was measured after harvesting on day at 27 and 32. After filtering through Whatman No. 4 filter paper (Whatman, Kent, UK), and washing with distilled water, the dry weights were measured after 48 h of freeze drying.

### Comprehensive metabolites profiling by GC-MS analysis

Twenty milligrams of each *L*. *paucicostata* sample treated with 1 μM coronatine was separately weighed, transferred into microfuge tubes (Axygen, Union City, CA, USA), and subjected to extraction with 1 mL of methanol (HPLC grade, Burdick & Jackson, Musketon, MI, USA). The samples were vortexed for 40 s and sonicated for 30 min. After sonication, the supernatants were collected separately from each sample and filtered through 0.45-μm polytetrafluoroethylene (PTFE) syringe filters (Membrane Solution, Plano, TX, USA). Two hundred microliters of each *L*. *paucicostata* plant sample was transferred into GC vials and dried with nitrogen gas for 5 min. For derivatization, 30 μL of 20,000 μg/mL methoxylamine hydrochloride in pyridine, 50 μL of BSTFA (N,O-bis (trimethylsilyl) trifluoroacetamide; Alfa Aesar, Ward Hill, MA, USA) containing 1% trimethylchlorosilane, and 10 μL of 3000 μg/mL myristic acid-*d*_27_ (Tokyo Chemical Industry Co., Ltd.) in pyridine used as an internal standard (IS), were added to the dried samples. The samples were incubated in a 65°C water bath for 60 min and analyzed by GC—MS.

The *L*. *paucicostata* plant samples were analyzed using an Agilent gas chromatography system (GC—MS, 7890A, Agilent Technologies, CA, USA) equipped with a model 5975C mass selective detector, a model 7683B series autosampler, a split/splitless injector, an injection module, and ChemStation software. The inlet temperature of GC was set to 250°C, and had an injection volume of 1.0 μL and a split ratio of 1:10. Helium was used as the carrier gas at a constant-flow rate of 1.0 mL/min. The detector voltage was set to 1553 V, and the auxiliary, MS source, and MS quadrupole temperatures were set to 280, 230, and 150°C, respectively. The mass range was set between 50 and 600 Da. A DB-5 MS column (Agilent Technologies) with 30 m × 0.25 mm I.D. × 0.25 μm d_f_ dimensions was used in the analysis. The initial oven temperature was set at 60°C and then ramped up to 310°C at 5°C/min. The total run time was 50 min.

The identification of metabolites in each sample was performed using the National Institute of Standards and Technology (NIST) mass spectral search program, and a match quality greater than 70% was used as the criterion for peak assignment. Additionally, the Human Metabolome Database (HMDB; http://www.hmdb.ca/) and Golm Metabolome Database (GMD; gmd.mpimp-golm.mpg.de/) were also used to identify the metabolites by comparison with the data. For relative quantification of metabolites, GC-MS spectrum data were processed using Expressionist^®^ MSX software (version 2013.0.39, Genedata, Basel, Switzerland), and a list of molecular features [retention time, intensity, and mass-to-charge ratio (m/z)] was obtained for each chromatogram. The normalization was then performed by dividing the intensity of each compound with that of the IS for relative quantification of the metabolites in each sample. Statistical analysis was performed by SPSS Statistics 23 software (IBM, Somers, NY, USA). Mann-Whitney test was employed to assess two independent groups (between groups of *L*. *paucicostata* under control and coronatine treatment at day 0). Comparisons of relative levels of comprehensive metabolites in three groups [control and the samples taken at day 27 (or day 32) after coronatine treatment at day 0, and day 23 (or day 28)] was assessed by the Kruskal-Wallis test, followed by Mann-Whitney test as post hoc analysis with Bonferroni’s correction.

### Absolute quantification of selected metabolites

The absolute quantification of *ρ*-coumaric acid, isoferulic acid, caffeic acid, sinapic acid, campesterol, and β-sitosterol was accomplished using calibration curves of each standard compound. Nine concentration solutions were prepared to achieve a concentration of 0.625 to 200 μg/mL by dissolving precisely weighed amounts of *ρ*-coumaric acid, isoferulic acid, caffeic acid, campesterol, and β-sitosterol in ethanol and sinapic acid in methanol. Two hundred microliters of each standard solution was transferred into GC vials and dried with nitrogen gas for 5 min. For derivatization, 30 μL of 20,000 μg/mL methoxylamine hydrochloride in pyridine, 50 μL of BSTFA (N,O-bis (trimethylsilyl) trifluoroacetamide; Alfa Aesar, Ward Hill, MA, USA) containing 1% trimethylchlorosilane, and 10 μL of 3000 μg/mL myristic acid-*d*_27_ (Tokyo Chemical Industry Co., Ltd.) in pyridine used as an internal standard (IS), were added to each standard compound of various concentrations. The samples were incubated in a 65°C water bath for 60 min, and the derivatized standard solutions of each compound were injected into the GC-MS in triplicate. The standard calibration curves were achieved by plotting concentration against intensity ratio between compounds and the internal standard. The means of the slopes (S) and standard deviation of the intercepts (σ) were calculated. The limit of detection (LOD) and the limit of quantification (LOQ) were calculated by following equations.

LOD=3.3×σ÷S

LOQ=10×σ÷S

## Results and discussion

### Effects of exogenous coronatine treatment on the growth of *L*. *paucicostata*

**Fig 1:**
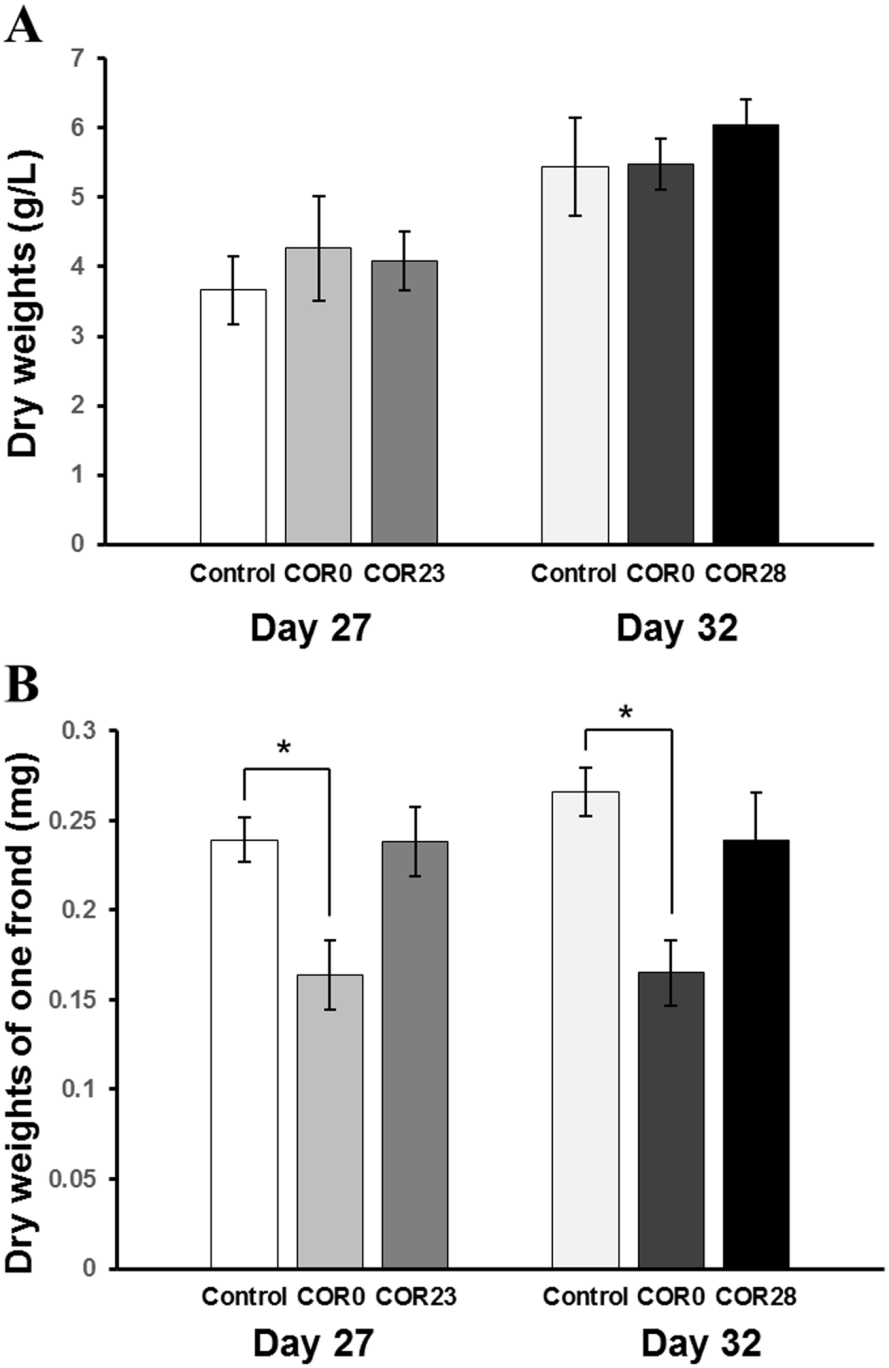
The growth of the whole *Lemna paucicostata* plants. Dry weight per liter (L: unit volume of culture medium) (A) and dry weight of one frond (B) of the whole *Lemna paucicostata* plants at day 27 and 32 under control and day 23 (COR23) and day 28 (COR28) under coronatine treatment. Bars indicate the mean values, and the error bars indicate the standard deviation (n = 4). Significant differences from the control group are indicated by asterisk based on the Mann-Whitney test (at a threshold of *p* < 0.05). COR0, coronatine treatment at day 0; COR23, coronatine treatment at day 23; COR28, coronatine treatment at day 28.

The effects of coronatine and elicitation timing on the growth of *L*. *paucicostata* were investigated. As shown in Fig 1A, there were no significant differences in the dry weight of whole plants of *L*. *paucicostata* with three coronatine treatment timings (day 0, 23, and 28) compared to the control. In previous studies, it was reported that 1 μM coronatine inhibited the growth of cultured *Vitis vinifera and Centella asiatica* cells and cotton seedlings [29,30,31], whereas the growth of *Taxus media* cell cultures treated with 1 μM coronatine showed similar pattern to that of un-treated culture [19]. The effects of coronatine treatment on the dry weights of one frond of *L*. *paucicostata* are shown in Fig 1B; these values were determined by dividing the total dry weights by the number of whole fronds of *L*. *paucicostata* (S2 Fig). The coronatine treatment on day 0 induced a statistically significant increase in the number of whole fronds at day 27 and 32 compared to that in the control. It can be inferred that the energy source was used to differentiate the plant cells and increase the number of whole fronds rather than to increase the dry weights of a single frond of *L*. *paucicostata* when coronatine treatment was applied on day 0. Consequently, it is suggested that elicitation of *L*. *paucicostata* using coronatine would be appropriate for obtaining useful bio-products without growth retardation.

### Effects of coronatine treatment on the metabolic profiles of *L*. *paucicostata* whole plant cultures

The effect of elicitation with coronatine on the comprehensive metabolic profiles of the whole plants of *L*. *paucicostata* was investigated, and the results are listed in Table 1. The metabolite identification levels of each compound in Table 1 are level 2 according to The Metabolomics Standards Initiative ([32] and S1 Table), except level 1 metabolites indicated by *. The relative levels of lysine, pyroglutamic acid, *ρ*-coumaric acid, campesterol, and β-sitosterol were the highest at day 27 in day 0 coronatine treated group. The highest levels of glycerol-3-phosphate, inositol phosphate, threonolactone and caffeic acid were observed on day 27 in day 23 coronatine treated group. Relatively higher levels of glycerol, erythronic acid, succinic acid, and sinapic acid were achieved on day 27 in both day 0 and day 23 coronatine-treated groups than in the control. Coronatine treatment at day 28 did not cause most of significant increase in the content of various metabolites. Significantly lower levels of asparagine, aspartic acid, glutamic acid, glutamine, ascorbic acid, fructose, and glucose were observed at day 27 in both day 0 and day 23 coronatine-treated groups than in the control. Significant decreases in the levels of various amino acids, such as asparagine, aspartic acid, glutamic acid, and glutamine, and carbohydrate compounds, such as fructose and glucose imply the more of these compounds were consumed for enhancement of the growth of the whole plants of *L*. *paucicostata* under the coronatine treated condition. The levels of succinic acid and malic acid, the major organic acids in the tricarboxylic acid (TCA) pathway, were significantly higher at day 27 and 32 under day 0 coronatine treatment conditions than their levels under the control condition.

**Table 1:** Relative intensities of various metabolites of *Lemna paucicostata* cultivated under coronatine elicitation conditions.

Compound	RT	Ion fragment (m/z)	TMS	Day 27			Day 32		
				Control	Coronatine treatment at day 0	Coronatine treatment at day 23	Control	Coronatine treatment at day 0	Coronatine treatment at day 28
**Alcohols**									
Glycerol	13.17	103, **205**, 218, 263	3	30.55±5.60^a^	68.18±9.38^b^	74.04±34.05^b^	18.55±2.56^A^	55.89±7.30^B^	30.51±9.52^C^
Glycerol-3-phosphate	24.93	299, **357**, 415, 445	4	71.06±12.9^a^	69.98±18.74^a^	91.24±8.24^b^	60.76±11.47	64.45±7.20	54.85±9.21
Inositol phosphate	35.80	217, 299, **315**, 470	7	40.97±10.78^a^	40.68±13.94^a^	58.42±6.29^b^	32.05±6.92^A^	42.55±5.82^B^	43.25±8.93^B^
Myo-inositol	31.28	**217**, 305, 381, 396	6	29.23±8.48	31.78±4.34	36.05±4.49	17.75±2.71^A^	24.42±2.54^B^	12.06±2.97^C^
**Amino acids**									
Alanine	11.55	**144**, 203, 218, 246	2	92.27±21.87	105.74±33.58	101.91±19.80	85.20±15.84^A^	65.00±13.40^AB^	66.54±8.21^B^
Asparagine	21.26	116, 130, **159**, 276	2	1878.04±243.40^a^	1299.77±165.88^b^	1291.24±71.80^b^	2178.30±254.53^A^	1532.63±211.79^B^	1879.35±184.08^C^
Aspartic acid	19.44	100, 218, **232**, 349	3	107.01±20.30^a^	73.78±10.47^b^	67.72±15.81^b^	97.43±29.99	70.99±11.76	82.12±9.43
Erythronic acid	20.39	205, 220, **292**, 409	4	20.46±3.70^a^	33.08±3.98^b^	37.59±8.22^b^	24.81±5.10^A^	38.06±3.92^B^	18.55±2.19^C^
Glutamic acid	21.79	128, 156, **246**, 363	3	396.89±40.03^a^	172.50±27.57^b^	245.40±51.94^c^	375.11±33.23^A^	140.18±11.78^B^	279.93±19.12^C^
Glutamine	25.19	**156**, 245, 347, 362	3	1107.78±106.35^a^	772.38±74.73^b^	857.79±48.06^b^	1210.70±74.26^A^	923.37±81.11^B^	1167.06±86.95^A^
Glycine	13.93	86, **174**, 248, 276	2	13.86±3.63	15.33±3.32	15.78±1.28	11.29±1.54	10.08±1.63	10.24±2.18
Isoleucine	13.63	**158**, 218, 232, 260	2	34.32±7.64	23.91±10.16	25.26±2.13	37.64±4.04^A^	25.25±2.58^B^	33.24±9.25^AB^
Lysine	28.18	**174**, 230, 317, 434	4	2.59±1.23^a^	4.72±1.34^b^	2.58±0.56^a^	2.13±0.70^A^	4.69±0.98^B^	1.36±0.52^A^
Phenylalanine	21.84	192, **218**, 266, 294	2	36.55±7.76	37.16±10.90	42.74±7.37	46.21±7.78	40.75±4.09	41.08±6.36
Serine	12.68	**116**, 132, 159, 188	2	109.82±26.76	90.32±30.49	75.60±15.64	91.44±23.74	77.62±17.54	50.50±22.33
Pyroglutamic acid	19.39	**156**, 230, 258, 273	2	540.71±100.70^a^	1018.53±273.15^b^	700.67±136.19^a^	605.57±103.81^A^	1447.88±431.71^A^	625.78±91.20^B^
Threonine	16.06	117, 203, **218**, 320	3	36.51±8.43^a^	22.29±11.07^b^	29.93±8.32^ab^	39.18±8.82^A^	23.87±5.99^B^	19.38±5.30^B^
Tyrosine	28.49	**218**, 280, 354, 382	3	24.40±2.57	20.78±7.12	25.43±5.50	25.18±3.10^AB^	20.58±2.30^A^	27.08±6.92^B^
Valine	8.24	**72**, 130, 156, 174	1	145.93±24.28	146.10±36.65	209.56±148.10	139.09±16.18	151.66±44.72	122.95±28.63
**Fatty acids**									
Glycerol monostearate	42.51	**399**, 412, 429, 487	2	21.49±4.49	18.12±4.79	23.91±2.79	25.25±3.52	22.21±2.76	26.08±3.32
Linoleic acid	33.58	**75**, 220, 262, 337	1	16.55±3.07	17.07±1.74	20.39±4.58	16.37±3.71^A^	17.98±1.89^A^	22.88±1.74^B^
α-Linolenic acid	33.69	**75**, 129, 335, 350	1	29.08±5.45^a^	34.46±3.73^ab^	46.76±15.38^b^	25.37±6.91^A^	38.73±5.68^B^	45.83±7.16^B^
1-Monopalmitin	39.70	239, 313, **371**, 459	2	36.41±6.83	33.14±7.39	39.31±3.91	46.81±4.95^A^	39.92±4.33^B^	46.97±5.81^AB^
Stearic acid	34.19	**117**, 145, 341, 356	1	7.80±1.82^ab^	7.31±0.57^a^	8.93±1.15^b^	6.26±0.41^A^	9.65±1.19^B^	6.41±0.63^A^
**Organic acids**									
Ascorbic acid	28.62	205, **332**, 449, 464	4	80.60±40.10^a^	28.13±8.21^b^	16.89±14.34^b^	24.28±16.03^A^	2.58±3.03^B^	11.16±4.42^A^
Fumaric acid	15.23	115, 133, 155, **245**	2	38.98±5.76	31.35±4.37	37.26±4.59	32.03±3.54	34.56±4.93	33.30±4.76
3-Hydroxymethylglutaric acid	21.35	115, 231, **247**, 363	3	16.72±9.92	11.23±3.47	10.35±4.69	22.84±11.63^A^	44.59±8.14^B^	24.71±8.34^A^
2-Keto-D-gluconic acid	25.27	217, **292**, 421, 511	5	8.68±0.80	11.32±2.51	8.92±1.37	8.48±1.14^A^	11.23±1.21^B^	7.06±1.01^A^
Malic acid	18.68	133, **233**, 245, 335	3	29.31±8.63^a^	53.46±4.58^b^	45.83±14.19^ab^	24.90±6.38^A^	49.07±5.19^B^	17.61±3.09^C^
Succinic acid	14.25	129, 218, **247**, 262	2	18.46±5.85^a^	30.44±5.52^b^	29.11±3.89^b^	17.80±4.35^A^	27.90±4.67^B^	15.29±2.45^A^
Threonolactone	15.71	116, 131, **247**, 262	2	1.68±0.44^a^	1.71±0.30^a^	2.53±0.50^b^	1.78±0.35	2.16±0.37	2.07±0.35
**Phenolics**									
Caffeic acid*	32.25	219, 307, 381, **396**	3	8.63±1.82^a^	10.73±1.87^a^	16.95±2.80^b^	10.06±1.70^A^	13.82±2.85^B^	7.46±1.18^C^
m-Coumaric acid	25.50	**219**, 249, 293, 308	2	2.37±0.44^a^	1.43±0.19^b^	1.94±0.15^a^	1.89±0.47	1.82±0.27	2.03±0.25
*ρ*-Coumaric acid*	28.54	**219**, 249, 293, 308	2	17.82±4.28^a^	24.17±3.63^b^	16.18±1.30^a^	19.13±3.12^A^	32.37±4.93^B^	16.85±1.39^A^
Isoferulic acid*	31.43	249, 308, 323, **338**	2	12.82±3.42^ab^	11.48±2.30^a^	17.52±2.75^b^	11.31±3.98^A^	17.32±3.78^B^	13.96±1.38^AB^
Sinapic acid*	34.12	323, 338, 358, **368**	2	0.81±0.26^a^	1.68±0.54^b^	1.32±0.19^b^	1.16±0.18^A^	3.28±0.84^B^	1.52±0.27^C^
**Phytosterols**									
Campesterol*	48.50	**129**, 343, 282, 472	1	7.13±0.99^a^	8.85±0.99^b^	7.35±0.80^a^	7.05±0.64^A^	9.47±1.37^B^	6.85±1.01^A^
β-Sitosterol*	49.53	**129**, 357, 396, 486	1	7.68±1.23^a^	11.01±2.35^b^	8.14±0.75^a^	8.75±1.62	9.99±1.76	8.61±1.31
Stigmasterol	48.80	55, **83**, 129, 484	1	18.02±2.75	15.60±1.87	16.15±2.41	17.91±1.30	20.31±3.05	18.23±1.80
**Sugars**									
Fructose	25.97	191, **204**, 217, 437	5	7707.92±1216.01^a^	4662.33±513.49^b^	5311.76±396.30^c^	6688.07±871.43^A^	3306.13±453.51^B^	3194.96±543.29^B^
Glucose	27.59	191, **204**, 217, 435	5	16125.74±2142.47^a^	10247.93±1200.77^b^	10333.66±1038.86^b^	13746.71±2176.35^A^	8224.34±892.56^B^	7286.62±963.43^B^
Glyceric acid	14.62	103, 133, **189**, 292	3	7.32±2.26	9.72±1.83	10.31±2.59	8.30±1.56^A^	8.34±0.83^A^	4.37±0.54^B^
Maltose	45.00	**204**, 217, 361, 451	8	7.06±1.89^a^	4.88±0.83^b^	9.95±4.65^a^	3.33±1.42^AB^	4.80±0.73^A^	3.64±0.53^B^
Sucrose	40.35	217, **361**, 437, 451	8	4387.74±695.69	3574.66±472.55	3859.37±326.21	5151.04±782.49^A^	3557.20±480.92^B^	4374.96±475.44^C^
**Others**									
γ -Aminobutyric acid	19.63	86, **174**, 304, 319	3	1293.49±128.29^a^	935.16±228.19^b^	1294.90±169.74^a^	1210.67±156.73^A^	761.16±121.85^B^	877.51±155.70^B^
Serotonin	37.62	**174**, 290, 449, 464	4	145.01±28.34^a^	76.86±23.43^b^	148.47±21.80^a^	168.72±31.60	130.25±19.78	160.22±24.55

Data are mean ± SD values of 8 measurements from four biological replications and duplicate analytical replications. The values were obtained by dividing the peak intensity of compounds by the peak intensity of the internal standard (myristic acid-*d*_27_).

Significant differences are indicated by different superscript letters based on the Kruskal-Wallis test; pairwise comparisons were made using Mann-Whitney test with Bonferroni’s correction (*p* < 0.017), and *p*-values for post hoc comparisons between groups of *L*. *paucicostata* are listed in S2 Table.

Lower case letters a—c indicate significant differences among three groups (control, coronatine treatment at day 0, and day 23) on day 27, and upper case letters A—C indicate significant differences among three groups (control, coronatine treatment at day 0, and day 28) on day 32.

The metabolite identification levels of each compound in this table are level 2 according to The Metabolomics Standards Initiative (MSI) [32], except level 1 metabolites indicated by *.

Base peak in each compound among ion fragments is shown as bold letters. Base peak means the highest ion fragments apart from ion fragments of TMS in various ion fragments of each compound, and it is used for relative quantification of each compound.

RT, retention time; TMS, trimethylsilylation.

*p*-Coumaric acid, isoferulic acid, caffeic acid, sinapic acid, campesterol, and β-sitosterol were selected among various metabolites identified by the comprehensive metabolic profiling, and quantification of those metabolites were performed. The regression equation, correlation coefficient (r^2^ values), the limit of detection (LOD) and the limit of quantification (LOQ) for the quantification of the selected phenolics (*p*-coumaric acid, isoferulic acid, caffeic acid, and sinapic acid) and phytosterols (campesterol and β-sitosterol) are indicated in Table 2. The productivity (P, mg/L) and daily productivity (DP, mg/L/day) of those compounds on day 27 and 32 in *L*. *paucicostata* treated with 1 μM coronatine on day 0 are listed in Table 3. The highest productivities and daily productivities of the phenolic compounds, *p*-coumaric acid (P: 1.75 mg/L, DP: 0.055 mg/L/day), isoferulic acid (P: 1.54 mg/L, DP: 0.048 mg/L/day), caffeic acid (P: 0.94 mg/L, DP: 0.029 mg/L/day), and sinapic acid (P: 0.82 mg/L, DP: 0.025 mg/L/day) were achieved on day 32 by coronatine treatment on day 0. In addition, the highest productivities and daily productivities of campesterol (P: 2.78 mg/L, DP: 0.087 mg/L/day) and β-sitosterol (P: 1.33 mg/L, DP: 0.042 mg/L/day) were also observed on day 32 by coronatine treatment on day 0.

**Table 2:** Regression equation, correlation coefficient (r^2^ values), LOD, and LOQ of standard compounds of phenolics and phytosterols.

Compounds	Regression equation	r^2^ values	LOD (μg/mL)	LOQ (μg/mL)
*ρ*-Coumaric acid	y = 0.0028x − 0.0072	0.9997	3.74	11.35
Isoferulic acid	y = 0.003x − 0.0201	0.9991	8.76	26.54
Caffeic acid	y = 0.0084x − 0.0499	0.9992	1.29	3.92
Sinapic acid	y = 0.0028x − 0.0152	0.999	2.58	7.83
Campesterol	y = 0.0007x − 0.0063	0.9926	2.62	7.94
β-Sitosterol	y = 0.002x − 0.0115	0.9993	4.45	13.49

Triplicate measurements were performed for each test.

**Table 3:** Productivity (mg/L) and daily productivity (mg/L/day) of selected phenolic compounds and phytosterols on days 27 and 32 in *Lemna paucicostata* culture upon 1 μM coronatine treatment.

Compound	Content	Day 27		Day 32	
		Control	Coronatine treatment at day 0	Control	Coronatine treatment at day 0
*ρ*-Coumaric acid	Productivity (mg/L)	0.75±0.22	1.08±0.23*	1.16±0.25	1.75±0.31*
	Daily productivity (mg/L/day)	0.028±0.0080	0.040±0.0086*	0.036±0.0077	0.055±0.0096*
Isoferulic acid	Productivity (mg/L)	0.91±0.20	1.01±0.20	1.28±0.22	1.54±0.23*
	Daily productivity (mg/L/day)	0.034±0.0075	0.038±0.0075	0.040±0.0068	0.048±0.0073*
Caffeic acid	Productivity (mg/L)	0.58±0.08	0.69±0.10	0.88±0.11	0.94±0.09
	Daily productivity (mg/L/day)	0.021±0.0031	0.026±0.0038	0.027±0.0033	0.029±0.0029
Sinapic acid	Productivity (mg/L)	0.47±0.06	0.58±0.10	0.72±0.09	0.82±0.08*
	Daily productivity (mg/L/day)	0.017±0.0022	0.021±0.0036	0.022±0.0028	0.025±0.0025*
Campesterol	Productivity (mg/L)	1.58±0.26	2.07±0.30*	2.34±0.29	2.78±0.33*
	Daily productivity (mg/L/day)	0.059±0.0096	0.077±0.011*	0.073±0.0092	0.087±0.010*
β-Sitosterol	Productivity (mg/L)	0.79±0.14	1.07±0.12*	1.24±0.18	1.33±0.17
	Daily productivity (mg/L/day)	0.029±0.0050	0.040±0.0046*	0.039±0.0057	0.042±0.0052

Data are mean ± SD values of 8 measurements from four biological replications and duplicate analytical replications.

Asterisk (*) indicates statistically significant differences from the control, as analyzed by the Mann-Whitney test.

Caffeic acid, sinapic acid, and *ρ*-coumaric acid belong to hydroxycinnamic acid group. Caffeic acid and its derivatives are known to possess antioxidant, antiinflammatory, antiviral, and immunostimulatory activities [33]. Caffeic acid phenethyl ester was shown to inhibit the growth of human leukemia HL-60 cells [34]. Ferulic acid produced by plants usually exists as a *trans*-isomer, namely isoferulic acid [35]. In a previous study, ferulic acid was shown to have antiinflammatory activity in RAW264.7 cells [36]. Sinapic acid derivatives have been found to be the most important phenolic compounds in rape seed and mustard oil, and in broccoli [37,38]. The antioxidative and antiinflammatory activities of sinapic acid and their ester derivatives have also been investigated previously [39,40]. *ρ*-Coumaric acid is known to possess antioxidant, anti-inflammatory, and antiplatelet activities [41]. Campesterol is an important structural component in the plant membrane [42], and it is known to possess LDL-cholesterol lowering activity in hypercholesterolemic human serum [43], and hepatic clearance capability in human [44]. β-Sitosterol is known to have anti-inflammatory, apoptosis inducing, chemopreventive, hypocholesterolemic, angiogenic anti-oxidative, and anti-diabetic activities [45]. β-Sitosterol also exhibits antimutagenic activities against tetracycline [46].

In a previous report, the coronatine showed a similar mechanism of action to the methyl jasmonate [47] with more effectiveness than methyl jasmonate for enhanced production of various taxanes [48]. Jasmonates are signaling molecules that activate several important physiological processes in various plants, and the biosynthesis of jasmonates, induced by external biological or non-biological stresses, triggers local and systematical defense responses [49]. Among various jasmonates, methyl jasmonate was reported to play an important role in signal transduction processes that regulate defense genes in plants [50], and exogenously treated methyl jasmonate enhanced various secondary metabolites including terpenoids, phenolic compounds, and alkaloids [51–53]. The exact target genes of coronatine treatment for the enhanced production of secondary metabolites are not clear in our study. However, it is speculated that various signal transduction processes might be modulated as defense response by the coronatine treatment in *L*. *puasicostata* plant. Elicitation with methyl jasmonate or silver nitrate in the whole plants of *L*. *paucicostata* reduced the production of phenolic compounds, such as caffeic acid and cinnamic acid, but enhanced the production of phytosterols [25]. However, coronatine treatment enhanced the production of various phenolic compounds, such as caffeic acid, isoferulic acid, sinapic acid, *ρ*-coumaric acid, and two phytosterols (campesterol and β-sitosterol). These findings suggest that the whole plants of *L*. *paucicostata* cultivated with coronatine treatment had enhanced activities of both the phenylpropanoid (S3 Fig), and phytosterol biosynthetic pathways. *ρ*-Coumaric acid was reported to be biosynthesized by phenylalanine ammonia lyase (PAL) and coumarate 4-hydroxylase (C4H), which converts phenylalanine to cinnamic acid and then cinnamic acid to *p*-coumaric acid, respectively [54]. Bifunctional hydroxycinnamaldehyde dehydrogenase (REF1 protein) was also suggested as a key enzyme in the ferulic acid and sinapic acid biosynthesis and it oxidizes coniferaldehyde and sinapaldehyde [54]. It was also reported that the expression of the PAL gene was greatly enhanced by coronatine in the suspension culture of *Vitis vinifera* [28]. In addition, hydroxymethylglutaryl-CoA reductase (HMGR) was reported as the key enzyme for biosynthesis of phytosterols, including campesterol and β-sitosterol [55]. Thus, it is assumed that coronatine might act to upregulate the activities of PAL, C4H, REF1 protein, and HMGR in the whole plant cultures of *L*. *paucicostata*. This assumption drawn above should be verified by transcriptomic and proteomic approaches in further study.

## Conclusions

In the present study, the effects of different timings of elicitation with coronatine on the growth and metabolic profiles in *L*. *paucicostata* were investigated using GC-MS. The total dry weights of the whole plants of *L*. *paucicostata* were not significantly different between the coronatine treated and untreated groups. The treatment of *L*. *paucicostata* with coronatine induced changes in the levels of various metabolites, including alcohols, amino acids, fatty acids, organic acids, phenolics, phytosterols, and sugars. Notably, we demonstrate, for the first time, that the coronatine treatment enhanced the levels of caffeic acid, *ρ*-coumaric acid, isoferulic acid, sinapic acid, campesterol, and β-sitosterol in the *L*. *paucicostata* plant cultures. In addition, the highest production of these phenolics and phytosterols was achieved at day 32 under day 0 coronatine treatment condition. These results provide information of metabolic responses of *L*. *paucicostata* to coronatine treatment and would contribute to the enhanced production of useful metabolites in the culture. The information obtained in this study can be used for industrial and commercial usage of *L*. *paucicostata* as a valuable bioresource.

## Supporting information

S1 Fig Growth curve of *L*. *paucicostata* grown under control and coronatine treatment. Data are mean values, and the vertical bars indicate the standard deviation from four biological replications.
(TIF)

S2 Fig Fronds number of *L*. *paucicostata* cultured by coronatine treatment. The fronds number of the whole *Lemna paucicostata* plants at day 27 and 32 under control and day 23 (COR23) and day28 (COR28) under coronatine treatment. Bars indicate the mean values, and the error bars indicate the standard deviation (n = 4). Significant differences from the control group are indicated by asterisk based on the Mann-Whitney test (at a threshold of *p* < 0.05). COR0, coronatine treatment at day 0; COR23, coronatine treatment at day 23; COR28, coronatine treatment at day 28.
(TIF)

S3 Fig Metabolic profiles of phenylpropanoid pathway of *L*. *paucicostata* cultured by coronatine treatment. Metabolic changes of *L*. *paucicostata* among three groups (control, coronatine treatment at day 0, and day 23) on day 27 are presented in upper graphs, and metabolic changes of *L*. *paucicostata* among three groups (control, coronatine treatment at day 0, and day 28) on day 32 are presented in lower graphs. Data are mean ± SD values of 8 measurements from four biological replications and duplicate analytical replications. Bars indicate the mean values, and the error bars indicate the standard deviation (n = 8). Different small and capital letters represent statistically significant differences examined by the Kruskal-Wallis test; pairwise comparisons were made using Mann-Whitney test with Bonferroni correction [significance level 0.017 obtained by division of 0.05 by 3 (hypotheses)]. COR0, coronatine treatment at day 0; COR23, coronatine treatment at day 23; COR28, coronatine treatment at day 28.
(TIF)

S1 Table Metabolites list according to MSI (The Metabolomics Standards Initiative) criterion. (DOCX)

S2 Table *P*-values for post hoc comparisons between groups of *L*. *paucicostata* using the Mann-Whitney test at *p* = 0.017 significance level according to Bonferroni's method. G1, G2, and G3 present three groups (control: G1, coronatine treatment at day 0: G2, and day 23: G3) on day 27, and G4, G5, and G6 present three groups (control: G4, coronatine treatment at day 0: G5, and day 28: G6) on day 32.
(DOCX)
