# Supplementary figures and images for: Effects of coronatine elicitation on growth and metabolic profiles of Lemna paucicostata culture
Source: PLoS One. 2017 Nov 3;12(11):e0187622. doi: 10.1371/journal.pone.0187622 (PMC5669466; doi:10.1371/journal.pone.0187622)

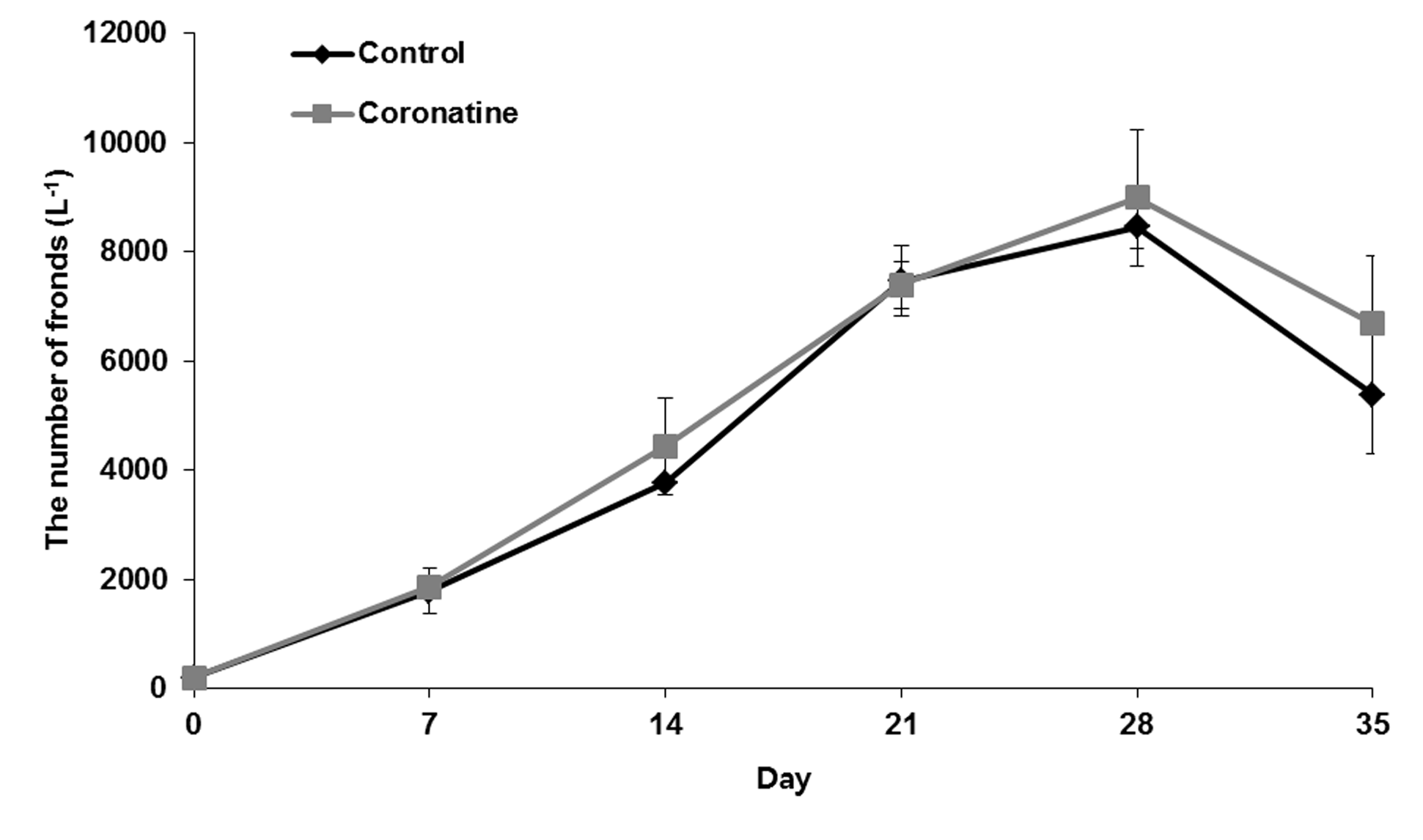

Supplement: S1 Fig — Data are mean values, and the vertical bars indicate the standard deviation from four biological replications. (TIF) [file pone.0187622.s001.tif]

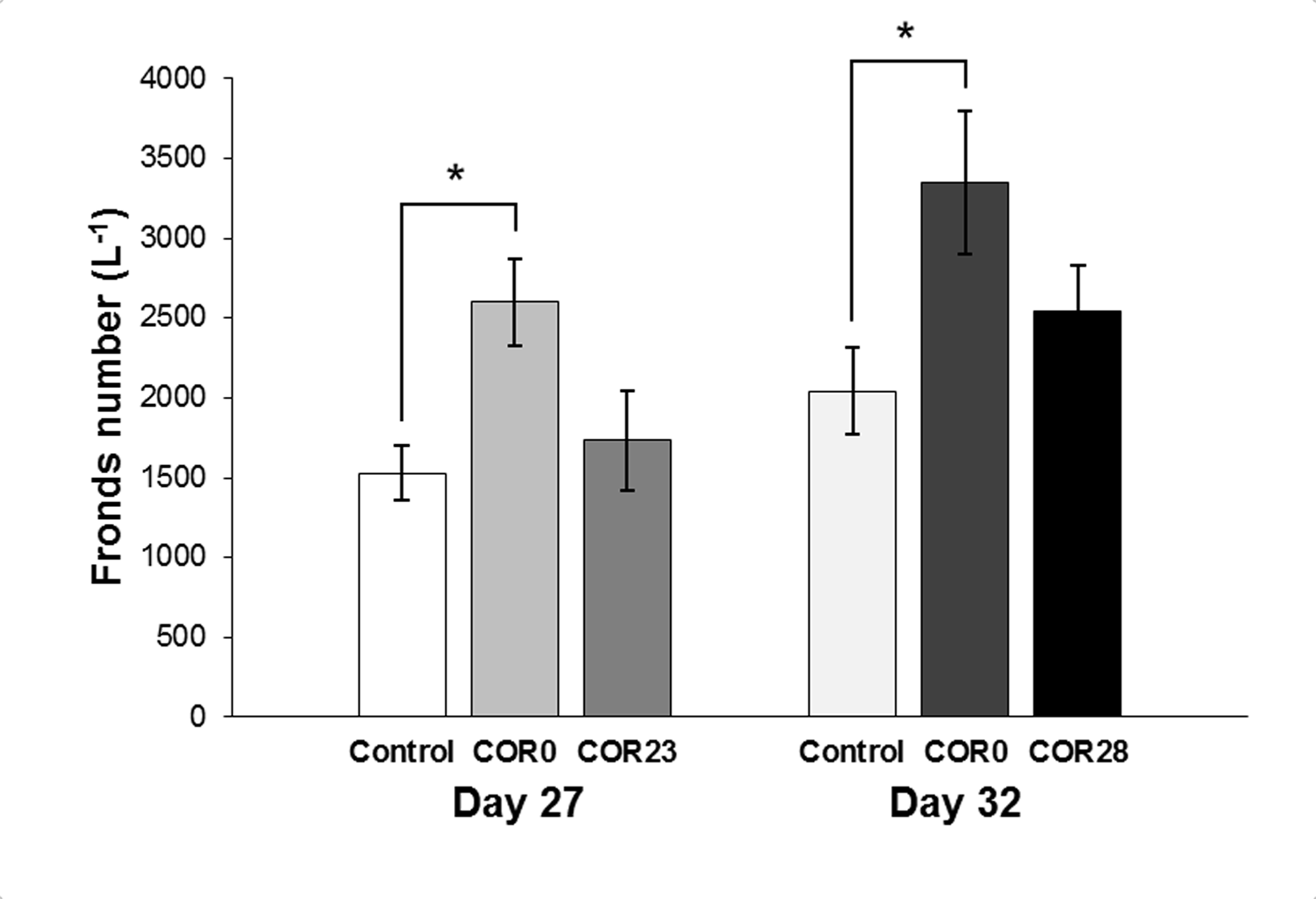

Supplement: S2 Fig — The fronds number of the whole Lemna paucicostata plants at day 27 and 32 under control and day 23 (COR23) and day28 (COR28) under coronatine treatment. Bars indicate the mean values, and the error bars indicate the standard deviation (n = 4). Significant differences from the control group are indicated by asterisk based on the Mann-Whitney test (at a threshold of p < 0.05). COR0, coronatine treatment at day 0; COR23, coronatine treatment at day 23; COR28, coronatine treatment at day 28. (TIF) [file pone.0187622.s002.tif]

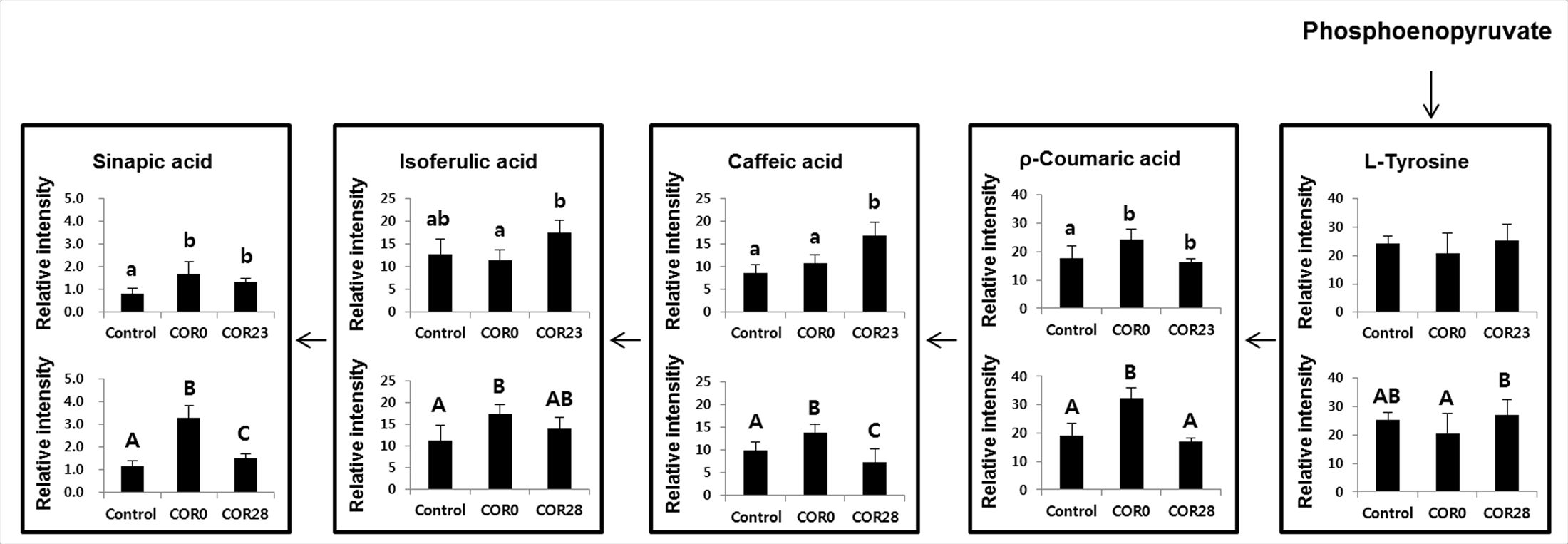

Supplement: S3 Fig — Metabolic changes of L. paucicostata among three groups (control, coronatine treatment at day 0, and day 23) on day 27 are presented in upper graphs, and metabolic changes of L. paucicostata among three groups (control, coronatine treatment at day 0, and day 28) on day 32 are presented in lower graphs. Data are mean ± SD values of 8 measurements from four biological replications and duplicate analytical replications. Bars indicate the mean values, and the error bars indicate the standard deviation (n = 8). Different small and capital letters represent statistically significant differences examined by the Kruskal-Wallis test; pairwise comparisons were made using Mann-Whitney test with Bonferroni correction [significance level 0.017 obtained by division of 0.05 by 3 (hypotheses)]. COR0, coronatine treatment at day 0; COR23, coronatine treatment at day 23; COR28, coronatine treatment at day 28. (TIF) [file pone.0187622.s003.tif]
