# Supplementary material for: Effects of coronatine elicitation on growth and metabolic profiles of Lemna paucicostata culture
Source: PLoS One. 2017 Nov 3;12(11):e0187622. doi: 10.1371/journal.pone.0187622 (PMC5669466; doi:10.1371/journal.pone.0187622)
Supplement: S1 Table — (DOCX) [file pone.0187622.s004.docx]

**S1 Table. Metabolites list according to MSI (The Metabolomics Standards Initiative) criterion.**

| **Compound** | **RT** | **Ion fragment (m/z)** | **TMS** |
| --- | --- | --- | --- |
|  |  |  |  |
| **Level 1^a^** | | |  |
| ρ-Coumaric acid | 28.54 | **219**, 249, 293, 308 | 2 |
| Isoferulic acid | 31.43 | 249, 308, 323, **338** | 2 |
| Caffeic acid | 32.25 | 219, 307, 381, **396** | 3 |
| Sinapic acid | 34.12 | 323, 338, 358, **368** | 2 |
| Campesterol | 48.50 | **129**, 343, 282, 472 | 1 |
| β-Sitosterol | 49.53 | **129**, 357, 396, 486 | 1 |
|  |  |  |  |
| **Level 2^b^** | | | |
| Valine | 8.24 | **72**, 130, 156, 174 | 1 |
| Alanine | 11.55 | **144**, 203, 218, 246 | 2 |
| Serine | 12.68 | **116**, 132, 159, 188 | 2 |
| Glycerol | 13.17 | 103, **205**, 218, 263 | 3 |
| Isoleucine | 13.63 | **158**, 218, 232, 260 | 2 |
| Glycine | 13.93 | 86, **174**, 248, 276 | 2 |
| Succinic acid | 14.25 | 129, 218, **247**, 262 | 2 |
| Glyceric acid | 14.62 | 103, 133, **189**, 292 | 3 |
| Fumaric acid | 15.23 | 115, 133, 155, **245** | 2 |
| Threonolactone | 15.71 | 116, 131, **247**, 262 | 2 |
| Threonine | 16.06 | 117, 203, **218**, 320 | 3 |
| Malic acid | 18.68 | 133, **233**, 245, 335 | 3 |
| Pyroglutamic acid | 19.39 | **156**, 230, 258, 273 | 2 |
| Aspartic acid | 19.44 | 100, 218, **232**, 349 | 3 |
| GABA | 19.63 | 86, **174**, 304, 319 | 3 |
| Erythronic acid | 20.39 | 205, 220, **292**, 409 | 4 |
| Asparagine | 21.26 | 116, 130, **159**, 276 | 2 |
| 3-Hydroxymethylglutaric acid | 21.35 | 115, 231, **247**, 363 | 3 |
| Glutamic acid | 21.79 | 128, 156, **246**, 363 | 3 |
| Phenylalanine | 21.84 | 192, **218**, 266, 294 | 2 |
| Glycerol-3-phosphate | 24.93 | 299, **357**, 415, 445 | 4 |
| Glutamine | 25.19 | **156**, 245, 347, 362 | 3 |
| 2-Keto-D-gluconic acid | 25.27 | 217, **292**, 421, 511 | 5 |
| m-Coumaric acid | 25.50 | **219**, 249, 293, 308 | 2 |
| Fructose | 25.97 | 191, **204**, 217, 437 | 5 |
| Glucose | 27.59 | 191, **204**, 217, 435 | 5 |
| Lysine | 28.18 | **174**, 230, 317, 434 | 4 |
| Tyrosine | 28.49 | **218**, 280, 354, 382 | 3 |
| Ascorbic acid | 28.62 | 205, **332**, 449, 464 | 4 |
| Myo-inositol | 31.28 | **217**, 305, 381, 396 | 6 |
| Linoleic acid | 33.58 | **75**, 220, 262, 337 | 1 |
| α-Linolenic acid | 33.69 | **75**, 129, 335, 350 | 1 |
| Stearic acid | 34.19 | **117**, 145, 341, 356 | 1 |
| Inositol phosphate | 35.80 | 217, 299, **315**, 470 | 7 |
| Serotonine | 37.62 | **174**, 290, 449, 464 | 4 |
| 1-Monopalmitin | 39.70 | 239, 313, **371**, 459 | 2 |
| Sucrose | 40.35 | 217, **361**, 437, 451 | 8 |
| Glycerol monostearate | 42.51 | **399**, 412, 429, 487 | 2 |
| Maltose | 45.00 | **204**, 217, 361, 451 | 8 |
| Stigmasterol | 48.80 | 55, **83**, 129, 484 | 1 |

Base peak among ion fragments is shown bold letters.

RT, retention time; TMS, trimethylsilylation

^a^Identified metabolites

^b^Putatively annotated compounds
