# Supplementary material for: Effects of coronatine elicitation on growth and metabolic profiles of Lemna paucicostata culture
Source: PLoS One. 2017 Nov 3;12(11):e0187622. doi: 10.1371/journal.pone.0187622 (PMC5669466; doi:10.1371/journal.pone.0187622)
Supplement: S2 Table — G1, G2, and G3 present three groups (control: G1, coronatine treatment at day 0: G2, and day 23: G3) on day 27, and G4, G5, and G6 present three groups (control: G4, coronatine treatment at day 0: G5, and day 28: G6) on day 32. (DOCX) [file pone.0187622.s005.docx]

**S2 Table. *P*-values for post hoc comparisons between groups of *L. paucicostata* using the Mann-Whitney test at *p* = 0.017 significance level according to Bonferroni's method. G1, G2, and G3 present three groups (control: G1, coronatine treatment at day 0: G2, and day 23: G3) on day 27, and G4, G5, and G6 present three groups (control: G4, coronatine treatment at day 0: G5, and day 28: G6) on day 32.**

| **Compound** | **Day 27** | | |  | **Day 32** | | |
| --- | --- | --- | --- | --- | --- | --- | --- |
|  | **G1 vs G2** | **G2 vs G3** | **G1 vs G3** |  | **G4 vs G5** | **G5 vs G6** | **G4 vs G6** |
| **Alcohols** |  |  |  |  |  |  |  |
| Glycerol | 0.000 | 0.959 | 0.000 |  | 0.000 | 0.001 | 0.005 |
| Glycerol-3-phosphate | 0.878 | 0.007 | 0.003 |  | 0.382 | 0.065 | 0.382 |
| Inositol phosphate | 0.959 | 0.010 | 0.010 |  | 0.005 | 0.721 | 0.015 |
| Myo-inositol | 0.645 | 0.065 | 0.161 |  | 0.001 | 0.000 | 0.005 |
|  |  |  |  |  |  |  |  |
| **Amino acids** |  |  |  |  |  |  |  |
| Alanine | 0.442 | 0.798 | 0.382 |  | 0.021 | 0.798 | 0.010 |
| Asparagine | 0.000 | 0.721 | 0.000 |  | 0.000 | 0.007 | 0.005 |
| Aspartic acid | 0.000 | 0.328 | 0.003 |  | 0.038 | 0.130 | 0.195 |
| Erythronic acid | 0.000 | 0.234 | 0.000 |  | 0.000 | 0.000 | 0.010 |
| Glutamic acid | 0.000 | 0.005 | 0.000 |  | 0.000 | 0.000 | 0.000 |
| Glutamine | 0.000 | 0.065 | 0.000 |  | 0.000 | 0.000 | 0.328 |
| Glycine | 0.574 | 0.721 | 0.050 |  | 0.195 | 0.959 | 0.382 |
| Isoleucine | 0.065 | 0.574 | 0.028 |  | 0.000 | 0.130 | 0.442 |
| Lysine | 0.005 | 0.001 | 0.328 |  | 0.000 | 0.000 | 0.028 |
| Phenylalanine | 0.878 | 0.105 | 0.130 |  | 0.195 | 0.959 | 0.161 |
| Serine | 0.234 | 0.328 | 0.021 |  | 0.279 | 0.015 | 0.005 |
| Pyroglutamic acid | 0.000 | 0.015 | 0.028 |  | 0.000 | 0.000 | 1.000 |
| Threonine | 0.015 | 0.161 | 0.279 |  | 0.003 | 0.130 | 0.000 |
| Tyrosine | 0.130 | 0.065 | 1.000 |  | 0.007 | 0.021 | 0.798 |
| Valine | 0.878 | 0.382 | 0.328 |  | 0.959 | 0.234 | 0.234 |
| **Fatty acids** |  |  |  |  |  |  |  |
| Glycerol monostearate | 0.161 | 0.028 | 0.328 |  | 0.083 | 0.028 | 0.442 |
| Linoleic acid | 0.959 | 0.083 | 0.083 |  | 0.382 | 0.000 | 0.000 |
| α-Linolenic acid | 0.038 | 0.028 | 0.002 |  | 0.002 | 0.028 | 0.000 |
| 1-Monopalmitin | 0.382 | 0.065 | 0.505 |  | 0.010 | 0.021 | 1.000 |
| Stearic acid | 0.721 | 0.007 | 0.083 |  | 0.000 | 0.000 | 0.959 |
|  |  |  |  |  |  |  |  |
| **Organic acids** |  |  |  |  |  |  |  |
| Ascorbic acid | 0.001 | 0.065 | 0.001 |  | 0.002 | 0.003 | 0.161 |
| Fumaric acid | 0.028 | 0.021 | 0.505 |  | 0.721 | 0.878 | 0.505 |
| 3-Hydroxymethylglutaric acid | 0.328 | 0.878 | 0.328 |  | 0.001 | 0.002 | 0.574 |
| 2-Keto-D-gluconic acid | 0.021 | 0.021 | 0.959 |  | 0.001 | 0.000 | 0.038 |
| Malic acid | 0.000 | 0.279 | 0.038 |  | 0.000 | 0.000 | 0.005 |
| Succinic acid | 0.002 | 0.279 | 0.002 |  | 0.001 | 0.000 | 0.195 |
| Threonolactone | 0.959 | 0.003 | 0.005 |  | 0.065 | 0.574 | 0.130 |
|  |  |  |  |  |  |  |  |
| **Phenolics** |  |  |  |  |  |  |  |
| Caffeic acid* | 0.050 | 0.001 | 0.000 |  | 0.015 | 0.000 | 0.007 |
| m-Coumaric acid | 0.000 | 0.000 | 0.021 |  | 0.959 | 0.130 | 0.442 |
| *ρ*-Coumaric acid* | 0.007 | 0.000 | 0.798 |  | 0.000 | 0.000 | 0.083 |
| Isoferulic acid* | 0.382 | 0.002 | 0.021 |  | 0.015 | 0.083 | 0.065 |
| Sinapic acid* | 0.001 | 0.279 | 0.002 |  | 0.000 | 0.000 | 0.005 |
|  |  |  |  |  |  |  |  |
| **Phytosterols** |  |  |  |  |  |  |  |
| Campesterol | 0.007 | 0.005 | 0.878 |  | 0.000 | 0.002 | 0.382 |
| β-Sitosterol* | 0.007 | 0.007 | 0.234 |  | 0.328 | 0.083 | 0.878 |
| Stigmasterol* | 0.083 | 0.798 | 0.161 |  | 0.130 | 0.195 | 0.645 |
| **Sugars** |  |  |  |  |  |  |  |
| Fructose | 0.000 | 0.010 | 0.000 |  | 0.000 | 0.721 | 0.000 |
| Glucose | 0.000 | 0.959 | 0.000 |  | 0.000 | 0.065 | 0.000 |
| Glyceric acid | 0.038 | 0.645 | 0.065 |  | 0.959 | 0.000 | 0.000 |
| Maltose | 0.010 | 0.000 | 0.195 |  | 0.028 | 0.002 | 0.328 |
| Sucrose | 0.021 | 0.195 | 0.065 |  | 0.000 | 0.010 | 0.010 |
|  |  |  |  |  |  |  |  |
| **Others** |  |  |  |  |  |  |  |
| γ -Aminobutyric acid | 0.005 | 0.005 | 0.721 |  | 0.001 | 0.161 | 0.002 |
| Serotonin | 0.001 | 0.001 | 0.878 |  | 0.028 | 0.021 | 0.798 |
